# Strengthening the Interactions Between Metal and Semiconductor Heterostructures via Microwave Synthesis for Chemiresistor Applications

**DOI:** 10.3390/nano15231786

**Published:** 2025-11-27

**Authors:** Rama Krishna Chava, Rajneesh Kumar Mishra

**Affiliations:** 1 Department of Chemistry, College of Natural Sciences, Yeungnam University, 280 Daehak-Ro, Gyeongsan 38541, Gyeongbuk, Republic of Korea; 2 Department of Physics, Yeungnam University, 280 Daehak-Ro, Gyeongsan 38541, Gyeongbuk, Republic of Korea

**Keywords:** core-shell nanoparticles, chemiresistors, schottky junctions, metal-semiconductor interactions, oxygen vacancies, H_2_ gas sensing

## Abstract

Designing metal–semiconductor-based core–shell nanostructures with strong interactions is emerging as a unique component for chemiresistor applications. Here, we have developed an effective hydrogen (H_2_) gas sensor based on Au-In_2_O_3_ core–shell nanostructures, which were synthesized through a short-time microwave hydrothermal process. At an optimal temperature of 375 °C, the device based on Au-In_2_O_3_ displays a high sensitivity of ~42, which is five times greater than that of the In_2_O_3_ toward 100 ppm of H_2_ gas. Moreover, the Au-In_2_O_3_ sensor showed higher selectivity toward H_2_ gas and stability over a long period. The excellent H_2_ gas-sensing performance of Au-In_2_O_3_ core–shell nanoparticles can be credited to the sensitization and Au catalytic effect core, and their strong interaction with the In_2_O_3_ component. Our work not only accounts for a facile synthesis approach for Au-In_2_O_3_ core–shell nanoparticles by synergistic properties of Schottky heterojunctions, but also offers a new insight into how strong metal–semiconductor interactions (SMSIs) play a dynamic role in developing high-performance gas-sensing devices.

## 1. Introduction

Hydrogen (H_2_) energy has recently been considered a promising alternative to solve environmental problems caused by fossil fuel energies. As an energy source, hydrogen has several benefits, such as it does not produce any pollution byproducts, is easily stored and delivered, prepared from water, and can be used in several areas, including domestic, industrial, and automotive [1,2]. However, hydrogen gas is highly flammable and explosive if the concentration is higher than 4% in air. In addition, hydrogen is tasteless, odorless, and colorless, hence it cannot be noticed by human senses [3,4]. Therefore, it is highly essential to develop techniques that make selective and accurate detection of hydrogen gas for safe purposes, especially in production areas, storage, and conveyance, which are all related to the hydrogen economy. For the quick detection of H_2_ gas, chemiresistors are among the simplest chemical sensor architectures due to their simple operation, low power consumption, portability, excellent miniaturization, quick measurement capability, and cost-effectiveness [5]. In 1992, the first chemiresistor was designed by Hughes and Schubert for H_2_ gas detection. After that, several researchers designed various chemiresistors based on metal oxide semiconductors (MOSs), which have possessed good sensing activity, but their higher operating temperature and durability are major drawbacks [6,7,8,9,10,11]. Therefore, the exploration of consistent, stable, and repeatable gas-sensing elements is a persistent endeavor with considerable promise from MOSs.

Indium oxide (In_2_O_3_) is an n-type semiconducting metal oxide with a direct bandgap of 3.5–3.7 eV, widely useful in chemiresistor applications because of its high conductivity, surface defects, good catalytic activity, low toxicity, and stability [12,13,14,15]. The gas-sensing performance of In_2_O_3_ depends on the electrical resistance changes due to the interaction of test gases and the In_2_O_3_ surface. Since the interaction of gas molecules mostly occurs at the surface, the gas response of In_2_O_3_ is meticulously connected to their morphology, grain size, and surface-to-volume ratios [16]. But pristine In_2_O_3_ nanostructures display poor sensitivity and selectivity, which restricts further practical applications.

To enrich the gas-sensing activity of In_2_O_3_ nanomaterials, fabrication of heterostructures with other semiconducting materials is a promising strategy. For example, In_2_O_3_-SnO_2_ [17], In_2_O_3_-WO_x_ [18], In_2_O_3_-ZnO [19], In_2_O_3_-rGO [20], In_2_O_3_-Co_3_O_4_ [21], and In_2_O_3_-NiO [22], etc., were prepared by several researchers, where heterostructures offered high gas response due to the synergic effects at the heterojunctions. In addition, the heterointerfaces could increase charge transduction, work function, and potential barrier modulation at the interfaces and surface properties such as defects and active sites, which are beneficial for sensing devices [23]. Another important and effective strategy to boost the sensing activity of In_2_O_3_ is a surface decoration with noble metal nanoparticles (NPs) such as Au [24], Ag [25], Pd [26], and Pt [27]. These noble metals improve the sensor activity toward the target gas by promotional effects, namely chemical sensitization and/or electronic sensitization processes. Here, the chemical sensitization occurs via the spill-over effect, which means the noble metal increases the sensor activity by increasing the reaction rate of chemical processes on the In_2_O_3_ surface. Next, electronic sensitization arises from the electronic interaction between two components at the interfaces of metal and In_2_O_3_ [28]. However, when the metal NPs are decorated on the In_2_O_3_ surface, they will suffer from undesired aggregation at moderate and higher operating temperatures, thereby losing their catalytic activity. In addition, the loaded metal NPs can be oxidized and adulterated by the gas byproducts, leading to the detachment from the In_2_O_3_ component [29,30,31]. Hence, to avoid the drawbacks of traditional approaches, metal–semiconductor core–shell heteronanostructures (CSHNSs) have gained more attention due to their exceptional properties in gas-sensing applications. These CSHNSs could offer improved specific surface area, synergistic properties, and stability. In addition, the metal NPs are protected by the semiconductor shell, which can prevent aggregation and delamination during the sensing activity [29,30,31,32]. Further, the CSHNSs also increase the electron transfer between the two components and thereby improve the gas-sensing properties. Therefore, it is important to fabricate a high-performance H_2_ gas-sensing device based on the CSHNSs.

In this regard, we prepared a novel H_2_ gas-sensing element, Au-In_2_O_3_ core–shell heteronanostructures. Different from conventionally tested hydrogen gas-sensing systems, the hybrid of metal Au NPs and semiconductor In_2_O_3_ nanostructures is designed as a core–shell system in two steps. Primarily, Au metal NPs were obtained by a citrate reduction method, and then an In_2_O_3_ shell was grown on the surface of Au NPs via a microwave hydrothermal process and followed by the calcination process. The suggested approach has many exceptional properties. First, Au NPs can be protected against aggregation due to the creation of a thick In_2_O_3_ shell on the surface of Au metal. Second, among precious metals, Au is promising for improving the gas-sensing properties since it exhibits excellent catalytic activity for oxidizing gases. Besides this, when Au is in contact with In_2_O_3_, a Schottky junction could be created, and an efficient electron transfer takes place. Third, the small-sized Au metallic NPs offer ideal adsorption sites for the test gas, from which activated species are spilled over to the In_2_O_3_ surface to react with the adsorbed oxygen ions. Lastly, the proposed microwave hydrothermal approach enables the Au-In_2_O_3_ CSHNSs with strong metal–semiconductor interactions, which not only avoids the aggregation of Au NPs, but also improves the electron transfer between Au and In_2_O_3_ components. With all these benefits, the Au-In_2_O_3_ CSHNSs show an excellent H_2_ gas-sensing response of 42, with good response and recovery times of 10 sec and 7 min, respectively, and also a high stability. At the end of this article, the reason for the improved H_2_ gas response of the Au-In_2_O_3_ sensor device compared to bare In_2_O_3_ and hydrothermally prepared Au-In_2_O_3_ CSHNSs is also discussed.

## 2. Materials and Methods

### 2.1. Chemicals and Reagents

All the chemicals used in the present work are of analytical grade. Indium tri-chloride (InCl_3_, 99.99%, Aldrich, Burlington, MA, USA), trisodium citrate dihydrate (C_6_H_5_Na_3_O_7_·2H_2_O, 98%, Showa Chemicals, Tokyo, Japan), HAuCl_4_·4H_2_O, 99%, Showa Chemicals, Tokyo, Japan, and urea (NH_2_CONH_2_, 98% Aldrich) were procured for the synthesis procedures. For all solution preparations, double-distilled water from a Milli-Q water purification system was utilized.

### 2.2. Preparation of Au-In_2_O_3_ Core–Shell HNSs

Au metal NPs with a 15 nm size were synthesized by using the trisodium citrate reduction method [33], and the detailed synthesis procedure is provided in the supporting information. To make Au-In_2_O_3_ Core–Shell samples, take 5 mL of prepared Au NPs in a separate vial and keep on stirring for 5 min. A solution containing 2.4 mM trisodium citrate and 2.0 mM InCl_3_ was gently added and kept on stirring for 0.5 hr. After stirring, 2 mM of urea was added and stirred for another 0.5 hr. Finally, the mixed solution was transferred to a 100 mL Teflon liner and heated in the microwave oven (CEM, MARS 6) at 180 °C for one hour. The ramping time to reach 180 °C from 20 °C is 10 min. After completing the reaction, the product was washed with distilled water five times and then dried at 60 °C overnight and finally calcined at 400 °C for two hours (ramping rate of 2 degrees per minute) in an air atmosphere.

The physical characterization of samples and the gas sensor device fabrication details are provided in the supporting information file.

## 3. Results

### 3.1. Formation Mechanism of Au-In_2_O_3_ CSHNSs

**Scheme 1:**
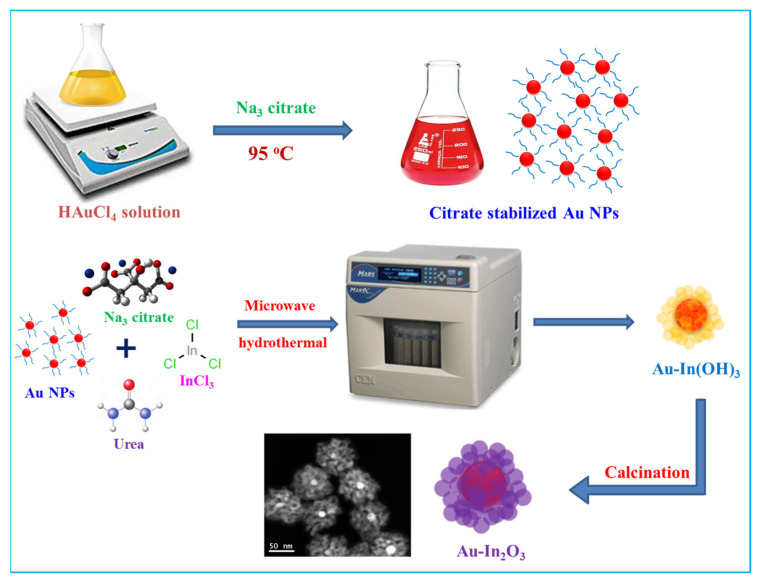
Detailed synthesis protocol for the preparation of Au NPs and Au-In_2_O_3_ core–shell NPs.

The fabrication of Au-In_2_O_3_ CSHNSs was attained by a two-step method in which metal Au NPs were first prepared by a sodium citrate reduction method, and then the microwave hydrothermal process was carried out to produce the In(OH)_3_ shell, resulting in the Au@In(OH)_3_ core-shell nanoparticles (CSNPs). Next, the calcination of Au@In(OH)_3_ results in the formation of Au-In_2_O_3_. The detailed approach for the synthesis of Au@In_2_O_3_ core–shell HNSs is shown in Scheme 1. Citrate-stabilized Au NPs (~15 nm) were synthesized through the reduction of chloroauric acid by trisodium citrate at a temperature of ~95 °C for 15 min. The formation of the In(OH)_3_ shell is accomplished by a microwave hydrothermal reaction (at 180 °C) with the precursor solution. When Indium chloride is dissolved in the deionized water, it produces In^3+^ ions, and these are easily attached to citrate ions. During the microwave hydrothermal reaction, urea worked as a pH buffer by gradually releasing the OH^−^ ions through the thermal decomposition, and then reacted with pre-formed In^3+^ ions to form In(OH)_3_ nanoparticles. In(OH)_3_ nanoparticles were slowly formed and stabilized in the solution due to the adsorption of citrate ions on the positively charged In^3+^ ions. This reaction simultaneously produces a number of In(OH)_3_ nanoparticles; these nanoparticles specially aggregated and self-assembled onto the Au NPs surface for the minimization of the total energy of the system [34,35,36]. After calcination at 400 °C, the formed Au@In(OH)_3_ core–shell HNSs will be transformed to the Au-In_2_O_3_ core–shell HNSs without changing the shape of core–shell particles.

### 3.2. Morphological and Structural Studies

**Figure 1:**
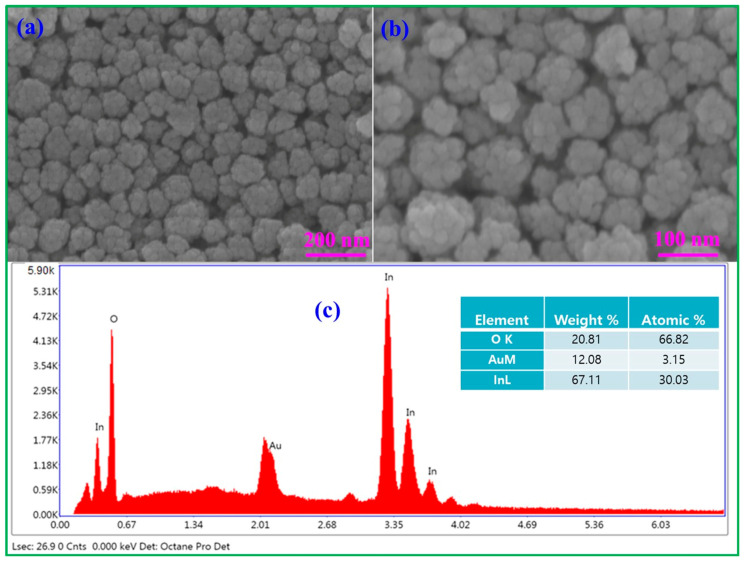
FE-SEM images (**a**,**b**) and EDAX profile (**c**) of Au@In_2_O_3_ CSHNSs.

**Figure 2:**
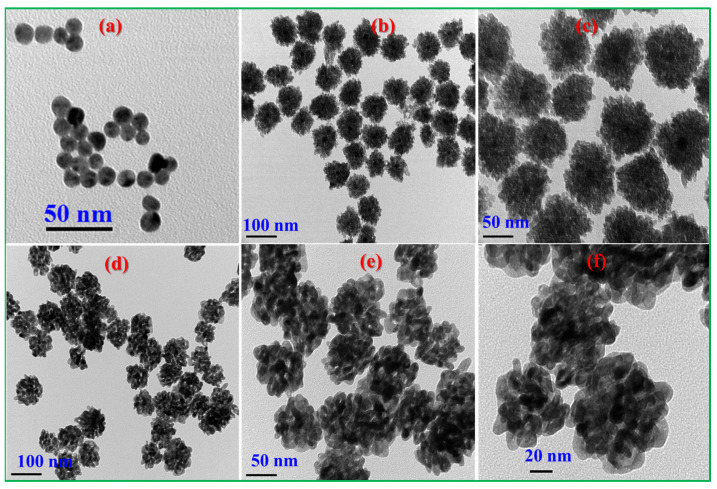
TEM images of (**a**) citrate-stabilized Au NPs, (**b**,**c**) Au@In(OH)_3_, and (**d**–**f**) Au@In_2_O_3_ core–shell NPs.

The surface morphology of Au-In_2_O_3_ CSHNSs was studied by taking FE-SEM images. As shown in Figure 1a,b, the synthesized Au-In_2_O_3_ possessed spherical-shaped nanostructures in which In_2_O_3_ NPs were aggregated onto the surface of Au NPs. The diameter of the as-prepared CSNPs was around 100 nm. Additionally, the energy dispersive X-ray spectroscopy (EDS) and mapping profiles were recorded to detect the elemental distribution of Au-In_2_O_3_ CSNPs, and the results are shown in Figure 1c and Figure S1, respectively. These EDS and mapping profiles revealed that the Au, In, and O elements co-existed in the prepared sample, and no other impurity peaks were detected. Further, the morphologies of the as-synthesized CSHNSs were also examined by TEM images. Figure 2a shows the TEM image of Au NPs having a diameter of below 15 nm.

In Figure 2b,c, the morphology of Au@In(OH)_3_ nanostructures was characterized by core–shell structures, in which Au NPs were observed as the core and an In(OH)_3_ shell was grown on the surface of Au NPs. After calcination, the Au@In(OH)_3_ CSNPs were converted into Au@In_2_O_3_ CSNPs, which are displayed in Figure 2d–f. A close observation of Figure 2f suggests that the shell comprises numerous In_2_O_3_ nanoparticles, and the size of the final Au@In_2_O_3_ CSNP was around 100 nm.

**Figure 3:**
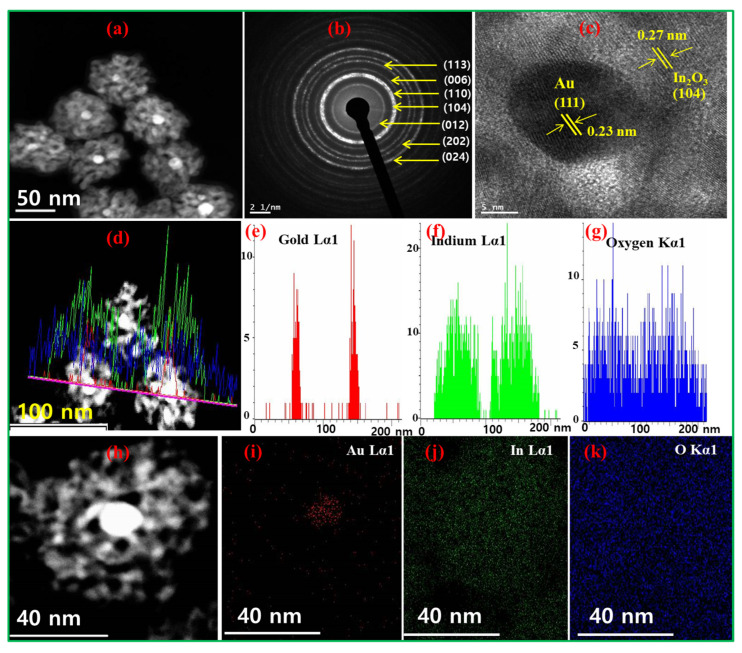
(**a**) STEM-HAADF, (**b**) SAED, (**c**) HR-TEM image of Au@In_2_O_3_ CSNP, which depicts the lattice spacings of both Au and In_2_O_3_ components, (**d**–**g**) line scanning profiles, and (**h**–**k**) STEM-EDS elemental mapping details of Au@In_2_O_3_ CSNPs.

To distinguish the Au core and In_2_O_3_ shell, the STEM-HAADF image is presented in Figure 3a. As seen from Figure 3a, the bright metal cores were Au NPs, whereas the surrounding shell was In_2_O_3_. Figure 3b shows the SAED patterns of Au@In_2_O_3_ CSNPs, in which the bright circular fringes are characteristic crystalline planes of In_2_O_3_. The heterojunctions between Au and In_2_O_3_ components were studied by HR-TEM image (Figure 3c), in which the lattice spacings for Au (111) and In_2_O_3_ (104) planes were determined as 0.23 and 0.27 nm, respectively. Next, Figure 3d–g present the STEM-EDS line scanning profiles, and these results confirmed the formation of Au@In_2_O_3_ CSNPs. In addition, STEM-EDS elemental mapping profiles (Figure 3h–k) also revealed that the core is made of Au NPs and the shell is made of In_2_O_3_ NPs. All these studies validate the formation of Au@In_2_O_3_ CSHNSs.

**Figure 4:**
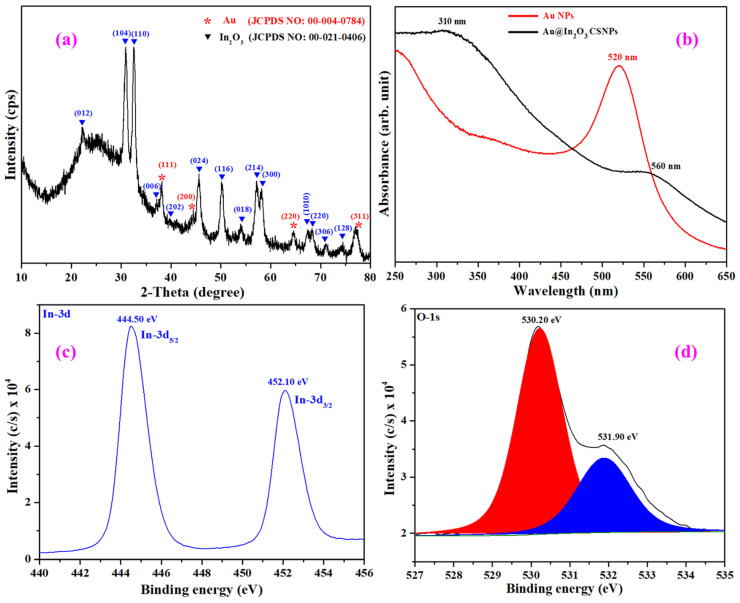
Powder X-ray diffraction patterns (**a**), UV-vis absorbance spectra of Au NPs and Au@In_2_O_3_ CSNPs (**b**); high-resolution XPS spectrum of (**c**) In-3d, and (**d**) O-1s core-levels.

Figure 4a shows the powder XRD patterns of as-synthesized Au-In_2_O_3_ CSHNSs. From Figure 4a, it is observed that Au metal NPs exhibited diffraction peaks at 38.22°, 44.43°, 64.58°, and 77.55°, corresponding to (111), (200), (220), and (311), respectively, of diffraction planes of Au with a cubic structure (JCPDS No: 00-004-0784). Meanwhile, strong diffraction peaks located at 22.32°, 30.96°, 32.59°, 37.14°, 39.95°, 45.64°, 50.24°, 54.12°, 57.20°, 58.21°, 67.43°, 68.32° and 71.02° corresponded to the planes of (012), (104), (110), (006), (202), (024), (116), (018), (214), (300), (1010), (220), (306) and (128), respectively, of In_2_O_3_ with the Rhombohedral structure (JCPDS No: 00-021-0406). No other peaks were identified in the diffraction patterns of Au-In_2_O_3,_ suggesting the purity of the prepared sample.

The formation of Au-In_2_O_3_ CSHNSs was further established by studying the UV-vis absorption spectra, as shown in Figure 4b. As seen from Figure 4b, the Au NPs exhibited a strong absorption peak at 520 nm, which is according to the surface plasmon resonance (SPR) peak of Au metal NPs. Figure 4b also shows the absorption spectrum of Au-In_2_O_3_ CSHNSs, which displayed two absorption bands around 310 nm and 560 nm; the first absorption band is attributed to the intrinsic absorption band of In_2_O_3_. The other absorption band around 560 nm arises from the SPR feature of the Au NP in the core–shell nanoparticles. After making the core–shell nanoparticles, it should be noted that the SPR peak is redshifted (~40 nm) due to the increase in the refractive index of the surrounding medium, and a similar phenomenon was observed in several core–shell nanoparticles [37,38,39].

Further, X-ray photoelectron spectroscopy analysis verifies the chemical states of constituent elements in the prepared core–shell nanostructures. The XPS survey spectrum (Figure S2) confirms the presence of In and O elements; however, the peaks related to the Au element were not well resolved, which might be due to the In_2_O_3_ shielding effect, and the same behavior was also supported by the reduced SPR peak intensity in Figure 4b. The relative atomic% of the In, O, and Au elements are calculated as 33.85, 66.01, and 0.14%, respectively. The high-resolution XPS of In-3d and O-1s core level spectra are provided in Figure 4. The binding energy values for the In-3d region (Figure 4c) were observed at 444.50 and 452.10 eV, which were assigned to In-3d_5/2_ and In-3d_3/2,_ respectively. It is also observed that the In-3d region is characterized by well-separated spin–orbit components (Δ = 7.6 eV), which confirms that the oxidation state of the indium element in the prepared core–shell nanostructures is +3 [14,40]. Next, Figure 4d displays the high-resolution XPS spectrum of O-1s, which is deconvoluted into three peaks at 530.0, 530.50, and 531.90 eV, and are noted as O_L_ (lattice oxygen), O_V_ (oxygen vacancies), and O_C_ (chemisorbed oxygen), respectively. It should be noted that the Au 4f XPS signal was not detected for the Au- In_2_O_3_ core–shell nanoparticles. This absence might be attributed to the thickness of the In_2_O_3_ shell or lower content of the Au core, which signals fall below the detection limit of the instrument.

### 3.3. Hydrogen Gas Sensing Properties of Microwave Hydrothermal Synthesized Au-In_2_O_3_ Core–Shell NPs

**Figure 5:**
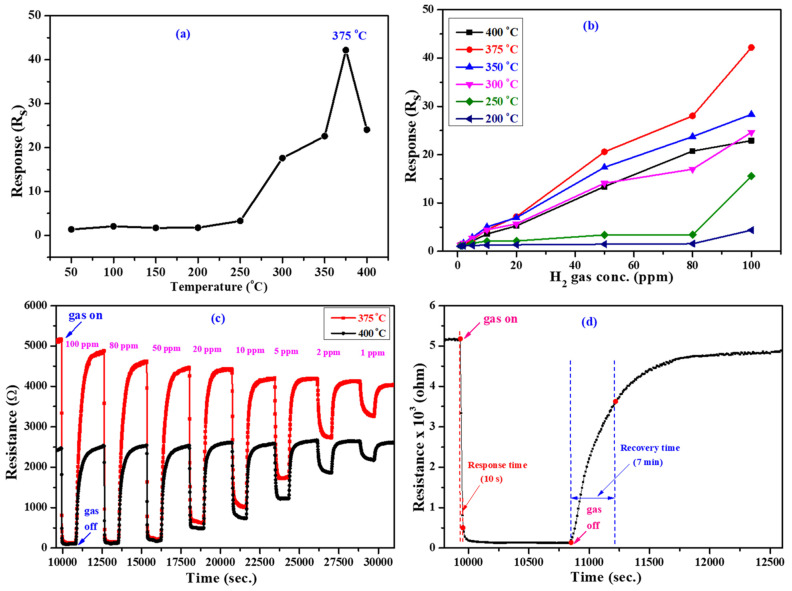
(**a**) H_2_ gas-sensing performance of Au@In_2_O_3_ gas sensor device (**a**) at different temperatures toward 100 ppm, (**b**) response at various temperatures toward 1–100 ppm H_2_ gas concentrations, (**c**) dynamic H_2_ gas-sensing response curves at 375 and 400 °C, and (**d**) response and recovery times.

To explore the advantages of Au-In_2_O_3_, we implemented the gas-sensing tests toward acetaldehyde, hydrogen, ethanol, nitrogen dioxide and carbon monoxide gases. The operating temperature is a substantial factor for metal oxide-based gas-sensing devices because it intensely affects the reaction to a testing gas. Therefore, the effect of temperature on the gas-sensing response of the Au-In_2_O_3_ device was tested at 100 ppm of H_2_ gas concentration, and the temperature was optimized. Figure 5a presents the corresponding curve of relative responses of the Au@In_2_O_3_ gas-sensing device at a temperature range of 50–400 °C. As shown in Figure 5a, as the temperature increases, the response also increases, and the highest response is achieved at 375 °C; hence, we chose 375 °C as the operating temperature for the successive gas-sensing measurements. The low responses below 375 °C are due to the lower surface activity, but as the temperature increases beyond a certain value, the response will diminish due to the accelerated desorption of the primarily adsorbed oxygen [41,42,43,44]. Figure 5a also reveals that the sensor device exhibited a response of ~42 at a temperature of 375 °C toward 100 ppm of H_2_ gas. Figure 5b shows the responses of the Au@In_2_O_3_ gas-sensing device at various temperatures ranging from 50 to 400 °C towards 1–100 ppm of H_2_ gas concentrations. It can be observed that the responses increase with the H_2_ gas concentration at all temperatures. Among them, the sensor device exhibited higher responses at 375 °C toward all gas concentrations. The responses of the Au-In_2_O_3_ gas sensor are 42.1, 28.0, 20.5, 7.1, 4.4, 2.4, 1.5, and 1.2 to 100, 80, 50, 20, 10, 5, 2, and 1 ppm, respectively, of H_2_ gas.

Therefore, the sensor device exhibits a good performance in terms of higher response at an operating temperature of 375 °C; hence, other gas-sensing measurements are mainly focused on the temperature mentioned above. From Figure 5b, it is also concluded that the Au-In_2_O_3_ core–shell NPs sensor also responded to lower temperatures, typically at 50 °C. Figure 5c shows the dynamic response curves of the as-prepared Au@In_2_O_3_ gas-sensing device for different concentrations (100–1 ppm) of H_2_ gas at 375 °C and 400 °C. When the sensor device is exposed to air, the resistance increases sharply and instantly. After the sensor contacts the H_2_ gas, the resistance decreases, and this specifies that the sensor exhibits n-type gas-sensing behavior. As seen from Figure 5c, the sensor device tested at 375 °C displayed a higher baseline resistance when compared to that at 400 °C, confirming that the device showed higher responses at 375 °C at all concentrations of H_2_ gas. It can be seen that the sensor displays a very fast response and recovery speed with gas on and off states. The sensor response also increases with increasing H_2_ gas concentration, demonstrating the outstanding detection ability for different gas concentrations. Next, Figure 5d presents the response and recovery times of the Au@In_2_O_3_ gas sensor. As mentioned in Figure 5d, the response and recovery times to 100 ppm H_2_ gas at 375 °C were determined as 10 s and 7 min, respectively.

**Figure 6:**
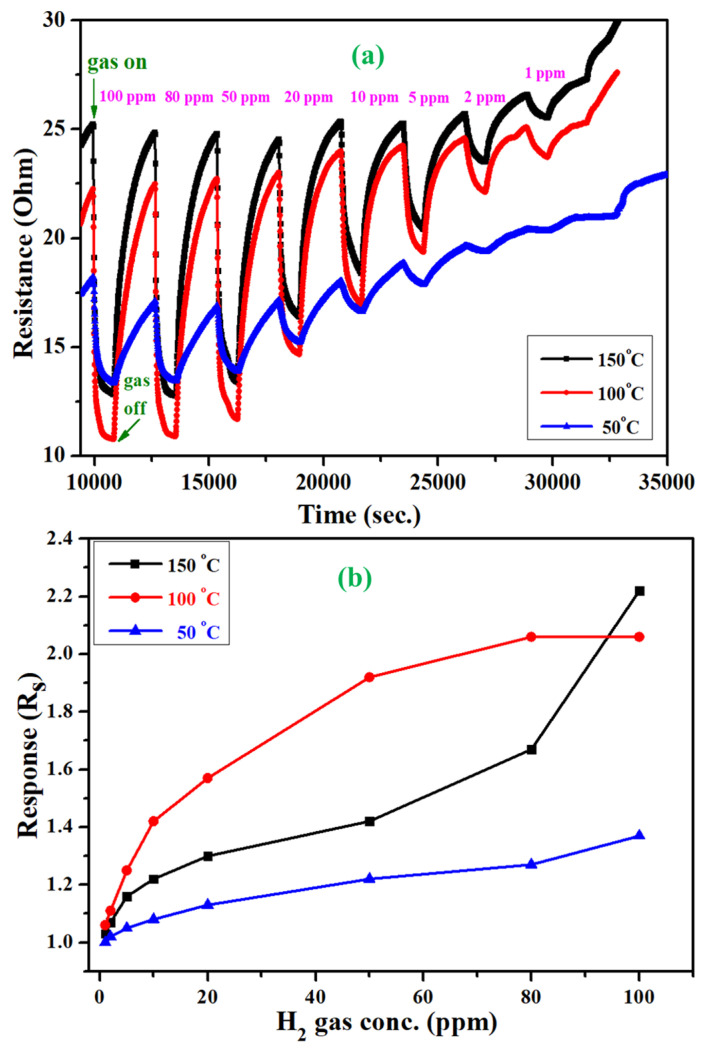
Dynamic gas-sensing response curves (**a**,**b**) response values of Au/In_2_O_3_ core–shell NPs tested at temeperatures of 150, 100 and 50 °C.

Of particular importance, the designed Au@In_2_O_3_ gas sensor device also displayed a significant, rapid response to low temperatures in the range of 50–150 degrees. During these measurements, with the increase of H_2_ gas concentration from 1 to 100 ppm, the sensor response also slightly increased from 1 to 1.37 at 50 °C and 1–2 at 100, and 150 °C. This response at lower temperatures is attributed to the formation of heterointerfaces between Au and In_2_O_3_ components. The continuous dynamic response and recovery curves of the H_2_ gas sensor based on Au@In_2_O_3_ were tested at 50, 100, and 150 °C (Figure 6a) upon exposure to hydrogen vapor at different concentrations from 1 to 100 ppm. As the hydrogen gas with a higher concentration was introduced into the testing chamber, the response of the sensor rapidly increased. As the gas supply is switched off, the responses almost recover to their initial value. In addition, the comprehensive responses of the Au@In_2_O_3_ sensor to H_2_ gas of different concentrations are summarized in Figure 6b.

**Figure 7:**
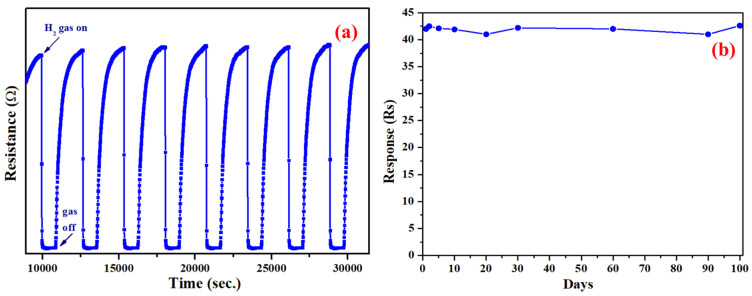
(**a**) Short-term and (**b**) long-term stability tests of Au-In_2_O_3_ CSHNSs toward 100 ppm of H_2_ gas.

Repeatability and stability are two significant parameters in evaluating the practical application of gas sensors in harmful and explosive gas environments. Therefore, the Au-In_2_O_3_ sensor device was tested at 100 ppm of H_2_ gas for nine successive cycles. As shown in Figure 7a, the sensor device shows response and recovery curves at 375 °C toward 100 ppm H_2_ gas. There were no significant changes in the sensing performance during the continuous cycles of the adsorption–desorption of H_2_ gas, confirming the excellent stability and repeatability of the Au-In_2_O_3_ sensor device. Further, the long-term stability of the Au/In_2_O_3_ sensor device was assessed by measuring the gas response separately after one week, one month, and three months in a dry atmosphere. As displayed in Figure 7b, the response sustained the initial value, suggesting that the Au-In_2_O_3_ core–shell NPs have sufficient stability, which extends the applications of Au-In_2_O_3_ core–shell NPs in industries. Table S1 summarizes the previous studies focused on metal–metal oxide core–shell nanostructured layers for sensing applications. Evidently, Au-In_2_O_3_ exhibits a superior and comparable H_2_ sensing performance to that of various core–shell structures.

### 3.4. H_2_ Gas-Sensing Mechanism over Au-In_2_O_3_ Core–Shell NP Surfaces

The sensing mechanism on the surface of metal oxides can be defined as the redox reaction between the surface-adsorbed oxygen molecules and exposed gas molecules, resulting in a change in the concentration of charge carriers. Due to the higher electron affinity of oxygen molecules, the oxygen molecule will capture electrons from the conduction band of indium oxide and form different oxygen species (O_2_^−^, O^−^, and O^2−^). The activated H_2_ molecules react with the oxygen species that are adsorbed on metal oxides, producing H_2_O, and releasing the electrons. In an air atmosphere, the oxygen molecules adsorb on the surface of the In_2_O_3_ sensor film and capture electrons, forming the oxygen ionic species O_2_^−^, O^−^, and O^2−^. The relevant reactions are noted as

(1) O_2 (gas)_ ↔ O_2 (ads)_

(2) O_2 (ads)_ + e^−^ → O_2_^−^

(3) O_2 (ads)_ + 2e^−^ → 2O^−^

(4) O_2 (ads)_ + 4e^−^ → 2O^2−^

Hence, an electron depletion layer is generated on the In_2_O_3_ surface_,_ and its electrical resistance increases. When the In_2_O_3_ sensor device is exposed to H_2_ gas, the chemisorbed oxygen species interact with H_2_ gas to generate water and release free electrons. These free electrons then flow back to In_2_O_3_; as a result, the electrical resistance of In_2_O_3_ decreases. These reactions are written as follows:

(5) H_2 (gas)_ ↔ H_2 (ads)_

(6) H_2 (ads)_ + O^−^ _(ads)_ → H_2_O_(gas)_ + e^−^

(7) H_2 (ads)_ +O^2−^ → H_2_O_(gas)_ + 2e^−^

**Figure 8:**
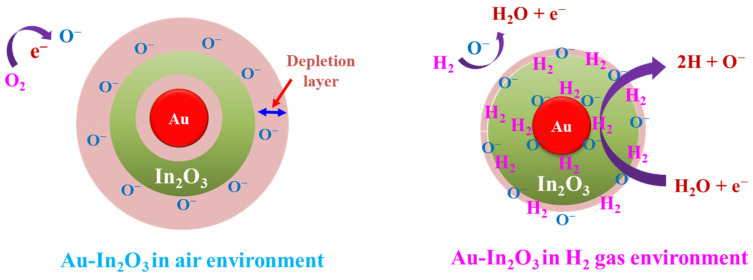
Behavior of depletion layer and gas-sensing mechanism of Au-In_2_O_3_ core–shell NPs in air and H_2_ gas exposures.

Based on the experimental results and discussions put together, overall, the improved gas-sensing response of Au-In_2_O_3_ core–shell nanostructures is credited to the following aspects. The Au NPs play a significant role in improving the response and selectivity of the Au-In_2_O_3_ core–shell HNSs. Due to the good catalytic nature, the electron-rich Au could enrich the process of catalytic oxidation and chemical sensitization by changing the surface energy via the spill-over effect [45]. As a result, the concentration of active oxygen species on the surface of the Au-In_2_O_3_ sensor device. Hence, the overall rate of adsorption–desorption of oxygen molecules on the sensors’ surface is improved. The electrons in the conduction band of In_2_O_3_ convert oxygen molecules into oxygen ions, which are exposed to the target gas (H_2_), release the electrons back to the Au-In_2_O_3_ sensor film via redox reaction, and improve the conductivity of the sensor. To start with the electronic sensitization effects, the formation of the Schottky junction between the Au metal and In_2_O_3_ components results in the transfer of electrons from In_2_O_3_ to Au metal, which acts as an electron sink [46]. Secondly, these metal Au NPs are good catalysts for oxygen dissociation and have high accessibility for catalyzing molecular oxygen dissociation [47]. The dissociated oxygen molecules could be absorbed on Au NP in the air to form the chemisorbed oxygen ions. Meanwhile, the chemisorbed oxygen ions produced on the Au could easily overflow to the In_2_O_3_ surface, resulting in more chemisorbed oxygen ions on the core–shell nanostructure surface. The detailed gas-sensing mechanism is presented in Figure 8.

### 3.5. Comparison of H_2_ Gas Sensing Properties of In_2_O_3_ and Au-In_2_O_3_ Core–Shell Nanostructures Synthesized via Hydrothermal and Microwave Methods

**Figure 9:**
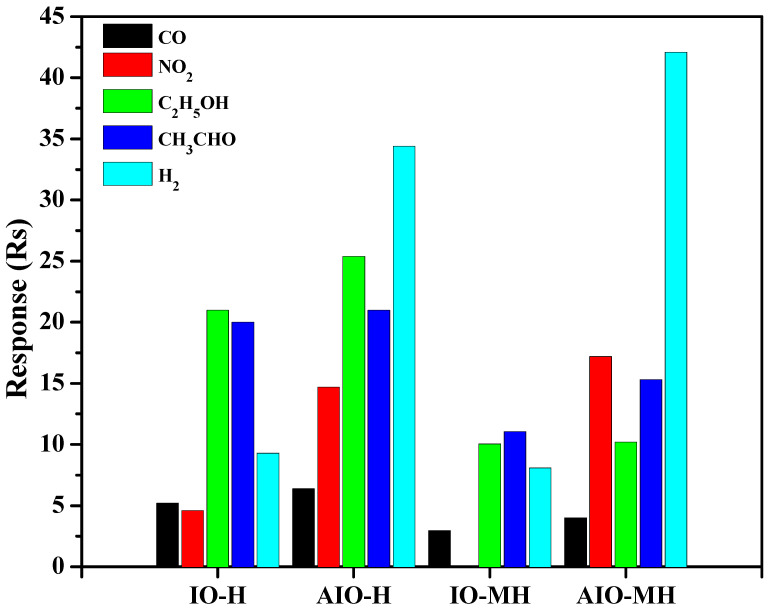
The investigation of responses and selectivities of the IO and AIO gas sensor devices prepared via hydrothermal and microwave hydrothermal methods. All sensing measurements were performed at 375 °C toward 100 ppm of test gases.

To understand the advantages of the microwave synthesis method and core–shell heteronanostructures, here we have attempted to compare the gas responses of other sensor devices based on bare In_2_O_3_ and Au-In_2_O_3_ core–shell samples, which were synthesized via the hydrothermal method [48]. For convenience, all the prepared samples are denoted as IO-H, AIO-H, IO-MH, and AIO-MH, where H refers to hydrothermal and MH refers to microwave hydrothermal methods. Figure 9 shows the gas-sensing performances and their selectivities of different sensor devices. It can be seen that gas-sensing performance was greatly improved between bare In_2_O_3_ and Au-In_2_O_3_ core–shell nanostructures, further Au metal NP significantly changes the selectivity of gases that were exposed, which makes the fabrication of core-shell heterostrucres is an attractive toward their implementation into gas sensing elements for industrial applications.

According to Figure 9, the sensor device based on the AIO-MH showed the highest gas response of 42.1, which is 4.5, 5.2, and 1.2 times greater than that of the IO-H, IO-MH, and AIO-H sensor devices toward 100 ppm of H_2_ gas. Selectivity is an essential factor in sensor devices and defines whether the device can work efficiently in a complex environment with multicomponent gases. Therefore, the sensor device was tested to 100 ppm of several gases such as acetaldehyde, ethanol, carbon monoxide, and nitrogen dioxide for selectivity performance. The selectivity toward H_2_ gas was achieved via the core–shell morphology of Au-In_2_O_3_ nanostructures. As given in Figure 9, all these gases show lower responses, suggesting that our device has great potential in discriminating against the H_2_ gas. Hence, the obtained results suggest that Au/In_2_O_3_ is an effective and stable active component for H_2_ gas sensors. Further, AIO-MH displayed an excellent and greater H_2_ gas-sensing response over the AIO-H, and the significant parameters that affected the gas response of AIO-MH over AIO-H are outlined as follows:

Firstly, the metal–semiconductor interactions were extensively studied to understand the charge transfer abilities between the two components. For this, the XPS characterization has been undertaken to examine the changes in the electron density on the surfaces of the Au metal core and In_2_O_3_ shell by investigating the shift in the binding energy values. If the electron transfers from one component’s surface to another, it can cause a change in its binding energy. Specifically, a decrease in the electron density results in a positive shift in binding energy, whereas a negative shift in binding energy values specifies an increase in the electron density [49]. According to Figure S3, the XPS of In-3d core level in AIO-MH sample displayed a negative shift in binding energy values when compared to IO-MH and AIO-H samples, depicting the electron-rich In_2_O_3_ surface, which in turn is accessible to the H_2_ sensing reactions. As a result, the AIO-MH sample exhibited a greater response toward H_2_ gas.

Next, oxygen vacancies and adsorbed oxygen species on metal oxide surfaces play important roles in gas-sensing applications [50,51]. Further oxygen vacancies are confirmed by X-ray photoelectron spectra, and could behave as additional electron donors, resulting in the enhanced H_2_ gas-sensing activity of AIO-MH samples. The high-resolution deconvoluted O-1s XPS spectra confirm the presence of a higher number of O-vacancies (O_v_) on the surface of AIO-MH (35.35%). In contrast, AIO-H core–shell nanostructures possess only 20.2% (Figure S4). In addition, the XPS spectra of O-1s reveal that the total concentration of O-vacancies and chemisorbed oxygen (O_v_ + O_c_) is around 70.06% in AIO-MH, whereas AIO-H only accounts for 65.15%, indicating that the higher number of O-species displayed, the greater response in the AIO-MH sensor device. Finally, oxygen vacancies are termed as active sites for oxygen gas molecule adsorption, and these O-vacancies expedite the creation of numerous chemisorbed oxygen species on the indium oxide surface [52]. The reaction between O_2_^-^ and H occurs effectively on the In_2_O_3_ surface, and as a result, a higher response was observed in AIO-MH core–shell nanostructures [53,54]. Additionally, the AIO-MH sensor device responds even at low temperature regions at 50–150 °C, signifying the importance of microwave-synthesized Au-In_2_O_3_ core–shell nanostructures for domestic and industrial applications. This low-temperature sensing activity is mainly due to the chemisorbed O_2_^-^ species present on the surface of In_2_O_3_. When a sensor device is exposed to H_2_ gas, the gas molecules react with O_2_^−^ species, releasing electrons to In_2_O_3_; consequently, the depletion layer is reduced, and the resistance decreases (O_2_^−^ + H_2_ → H_2_O + 2e). All these results indicate that the promotional (electronic and chemical sensitization) effects of Au NP and Schottky junction with In_2_O_3_ semiconductor; O-vacancies and strong interactions between Au and In_2_O_3_ enabled the efficient electron transportation and favored achieving the efficient H_2_ gas-sensing elements.

## 4. Conclusions

In this study, metal–semiconductor-based core–shell heterostructures composed of Au-In_2_O_3_ were synthesized to achieve selectivity and enhance the sensitivity toward H_2_ gas. The results show that the sample Au-In_2_O_3_ obtained from microwave hydrothermal reaction displayed the highest H_2_ gas response of ~42, which is 4.5, 5.2, and 1.2 times greater when compared to the bare In_2_O_3_, the hydrothermally prepared In_2_O_3,_ and Au-In_2_O_3_-based sensors. The synergetic effect of the strong metal–semiconductor interactions between Au and In_2_O_3_, and oxygen vacancies led to significantly enhanced H_2_ gas sensitivity, and even enabled the sensor to work at a lower temperature of 50 °C. Thus, microwave hydrothermal synthesized Au-In_2_O_3_ core–shell nanostructures can be widely applied as efficient sensing layers in the H_2_ monitoring industry.

## Data Availability

The data supporting the findings of this study have been included as part of the supporting information and are also available from the authors upon reasonable request.

## Supplementary Materials

The following supporting information can be downloaded at: https://www.mdpi.com/article/10.3390/nano15231786/s1. This file contains the synthesis of Au NPs, physical characterization of samples, gas-sensing device fabrication details, FE-SEM EDAX mapping profiles, and XPS spectra of In-3d and O-1s elements. Refs. [31,32,42,55,56,57,58,59,60,61,62] are cited in the supplementary material file.
