# Supplementary material for: Strengthening the Interactions Between Metal and Semiconductor Heterostructures via Microwave Synthesis for Chemiresistor Applications
Source: Nanomaterials (Basel). 2025 Nov 27;15(23):1786. doi: 10.3390/nano15231786 (PMC12693032; doi:10.3390/nano15231786)
Supplement: Supplementary file 1 [file nanomaterials-15-01786-s001.zip › nanomaterials-3989011-supplementary.pdf]

Strengthening the Interactions Between Metal and Semiconductor Heterostructures via Microwave Synthesis for Chemiresistor Applications

Rama Krishna Chava [1*] and and Rajneesh Kumar Mishra [2,*]

[1] Department of Chemistry, College of Natural Sciences, Yeungnam University, 280 Daehak-Ro, Gyeongsan 38541, Gyeongbuk, Republic of Korea

[2] Department of Physics, Yeungnam University, 280 Daehak-Ro, Gyeongsan 38541, Gyeongbuk, Republic of Korea

* Correspondence: rama@ynu.ac.kr (R.K.C.); rajneeshmishra@ynu.ac.kr (R.K.M.)

1. Synthesis of Au nanoparticles with a size of ~15 nm

A solution of trisodium citrate (25 ml, 34 mM) was added to $HAuCl_4$ solution (500 ml, 1mM) at boiling condition and the resulted solution was kept at that boiling temperature for 15 minutes with constant stirring. After cooling, take 5 ml of Au NPs in a separate vial and used for further experiment to grow indium oxide shell on their surface.

2. Physical characterization techniques

The morphology of synthesized nanostructures was studied by using Hitachi H-7600 transmission electron microscope (TEM) with an accelerating voltage of 100-400 kV. JEOL JEM-2200 FS field emission electron microscope outfitted with OXFORD INCA X-Sight detector was used to record the high resolution-TEM (HR-TEM) images, selected area electron diffraction (SAED) patterns, line scanning and elemental mapping analyses. Powder X-ray diffraction (XRD) patterns were collected using a PANalytical X-pert PRO diffractometer with Cu Kα radiation of 1.54060 Å. The shift in surface plasmon resonance peak of Au NPs was monitored by Shimadzu UV-2550 spectrophotometer in Ultraviolet-visible-near infrared (UV-vis-NIR) regions. The presence of constituent elements and their oxidation states in Au-In_2O_3 was investigated by X-ray photoelectron spectroscopy (Thermo Scientific K-Alpha instrument) with an Al Kα X-ray radiation source.

3. Gas sensor device fabrication and measurements details

For gas-sensing tests, Pt-interdigitated electrodes (1×1 cm^2) printed onto an alumina substrate (1.5×1.5 cm^2) was used as a chip for resistive based gas sensor measurements. Paste was made by mixing 0.03 g of microwave hydrothermal synthesized Au@In_2O_3 with 20 μl of α-terpinol (as a binder) and screen printed on the substrate. Subsequently, the sensor samples were aged at 400 ^oC for 2 h in air to stabilize the sensing layer. Gas sensing measurements were conducted in a sealed quartz tube furnace. The operating temperature was controlled by the electric furnace and varied from room temperature of 50-400 ^oC with testing gas concentrations of 2-100 ppm. The gas flow of N_2, Air and testing gas (H_2) was controlled by UNIT Instruments Inc. made high performance mass flow controller (MFC). The sensing element was connected to an external electrical circuit and the changes in the electrical resistance during adsorption and desorption of gas molecules on the material surface is estimated by measuring the electrical resistance in presence of air (R_a) and in gas (R_g). Humid air was generated by passing dry air through water, with the relative humidity (RH%) controlled by adjusting the air flow rate and maintained RH is about 10%. In general, as the RH% increases, the baseline resistance of the sensor decreases, leading to a decrease in its response to hydrogen. This behavior is due to the water molecules will form hydroxyl groups on the active sites. As a result, the concentration of chemisorbed oxygen species decreases, and hence response decreases. In this measurement, a computerized Agilent 34970A data acquisition/switch unit was used. The sensor response (S) was calculated as $S=R_g/R_a$ for an oxidizing gas or $S=R_a/R_g$ for reducing gas. Here R_a and R_g are the resistances of the sensor devise when subjected to air and target gas respectively. The response time was defined as the time required for the variation in the resistance to reach 90% of the equilibrium value after a test gas was injected, and the recovery time was the time necessary for the sensor to return to 10% above the original resistance in air after releasing the test gas.

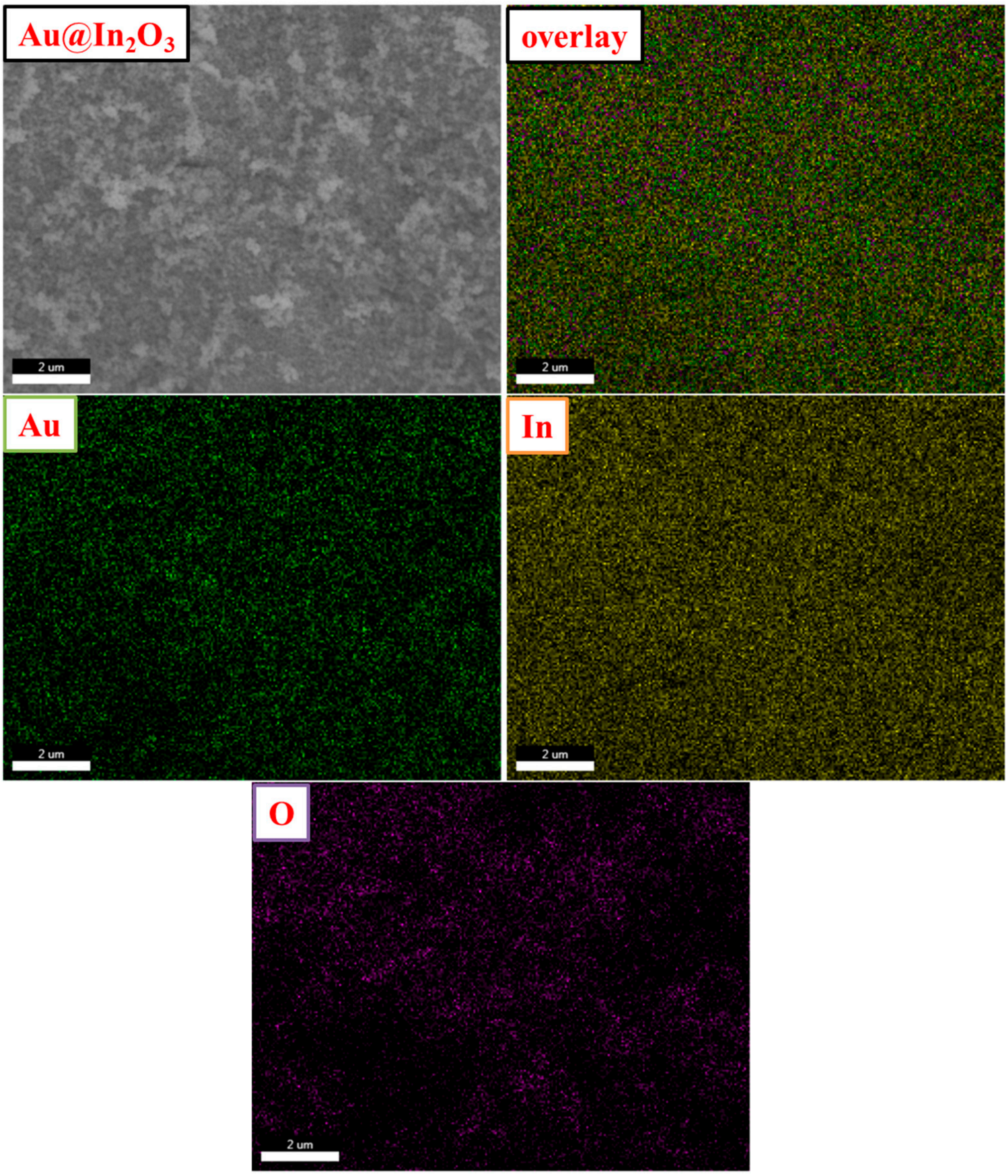

Figure S1. FE-SEM EDS mapping profiles of Au-In$_2$O$_3$ CSNPs.

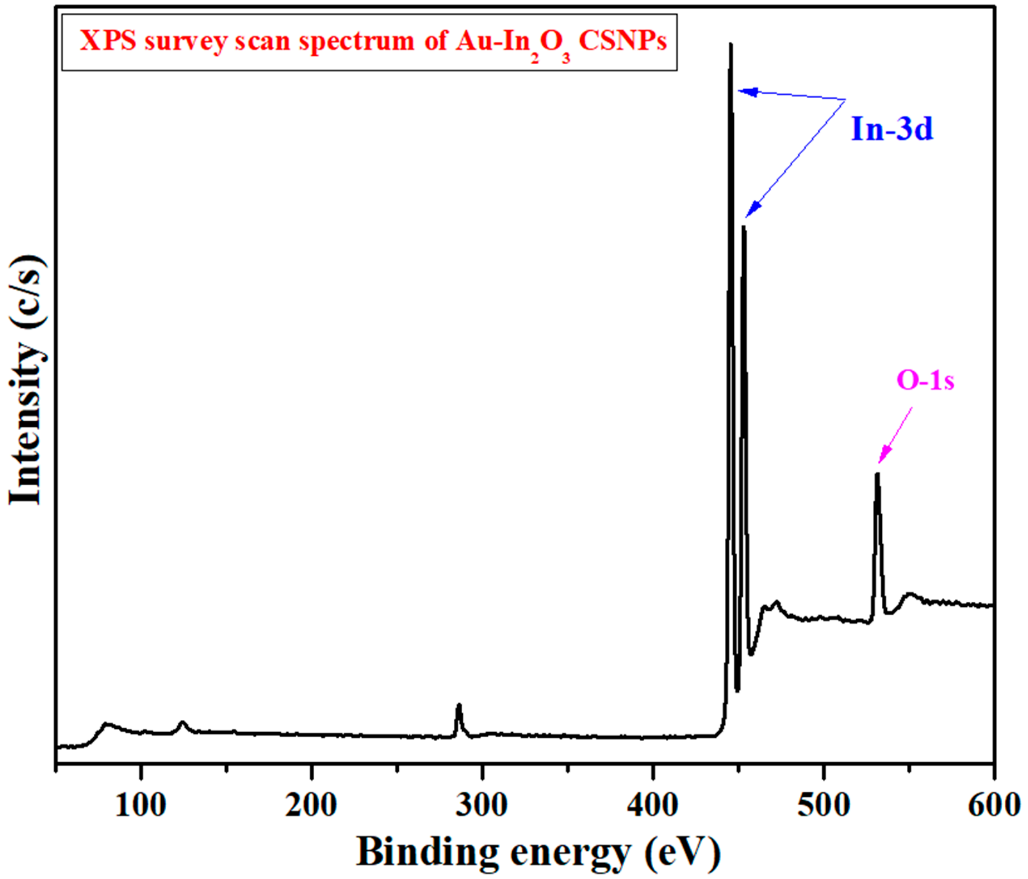

Figure S2. XPS survey scan spectrum of microwave hydrothermal synthesized Au-In$_2$O$_3$ cores-hell NPs

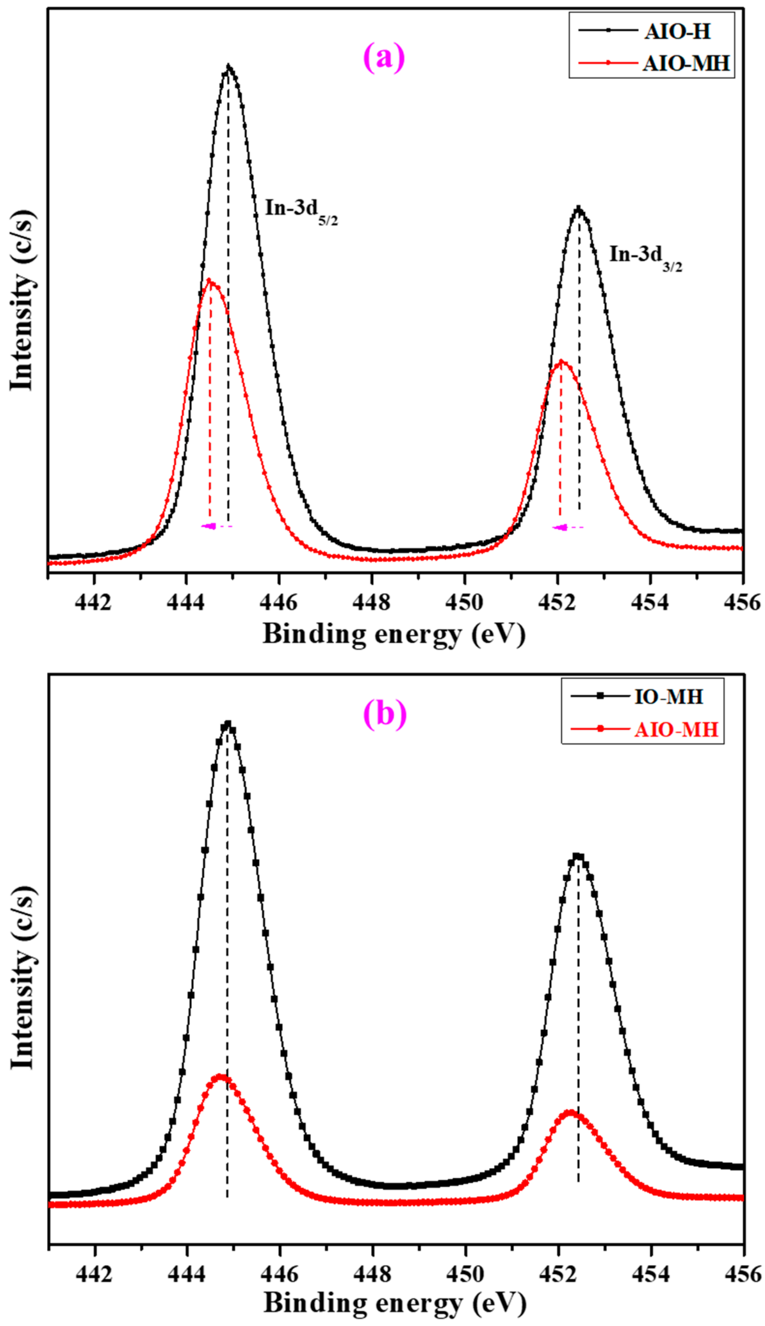

Figure S3. Understanding the XPS binding energy shift and comparison of In-3d core-level spectra of (a) AIO-H and AIO-MH; (b) IO-MH and AIO-MH samples.

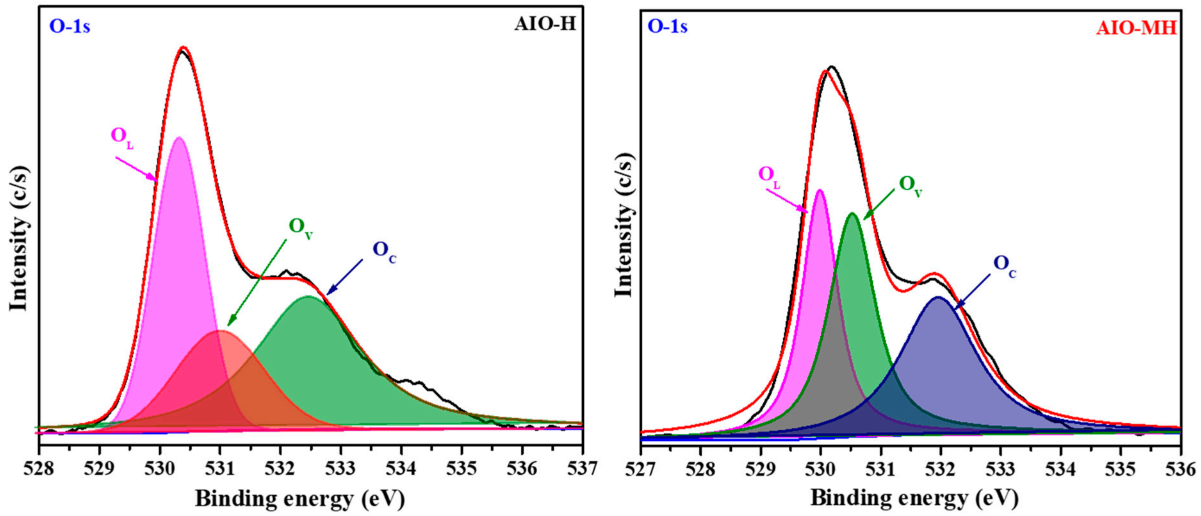

Figure S4. High-resolution deconvoluted XPS spectra of O-1s in AIO-H and AIO-MH core-shell nanostructures.

Table S1: A comparison table for gas sensing activities of different metal-metal oxide core-shell nanostructures

Core–shell NPs	Operating temp. (°C)	Target gas	Gas conc. (ppm)	Response (Rs)	Ref.
Au@ZnO	300 °C	Acetone	100	37	01
Au@TiO_2	600 °C	CO	1000	1.21	02
Pd@ZnO	350 °C	H_2	100	22	03
PdAualloy@ZnO	300 °C	H_2	100	80	04
Au/Co_3O_4/$W_{18}O_{49}$	270 °C	Triethylamine	2	16.7	05
Ag@In_2O_3	220 °C	Ethanol	50	72	06
Au@NiO	200 °C	Ethanol	100	2.54	07
Au@Co_3O_4	250 °C	Acetone	10	27	08
Pt@NiO	RT	H_2	5000	4.25	09
Pd@PdO	RT	H_2	10	5.85	10
Au@WO_3	100 °C	NO_2	1	20	11
Au@In_2O_3	375 °C	H_2	100	42	This work

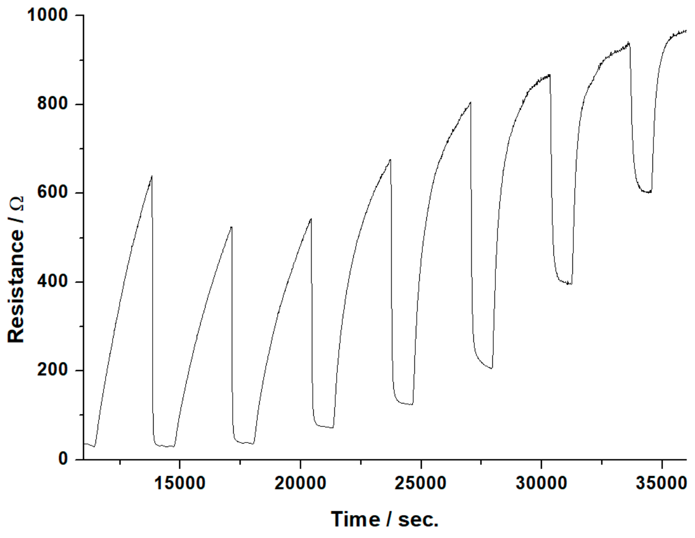

Figure S5. Dynamic response curves of Au-In_2O_3 cores-shell NPs subjected to ethanol gas at 375 ^{o}C toward 100-1 ppm gas.

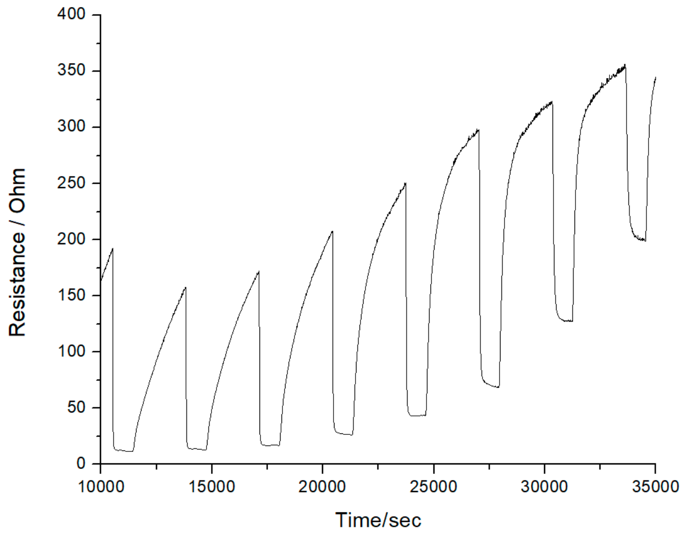

Figure S6. Dynamic response curves of Au-In_2O_3 cores-shell NPs subjected to acetaldehyde gas at 375 ^{o}C toward 100-1 ppm gas

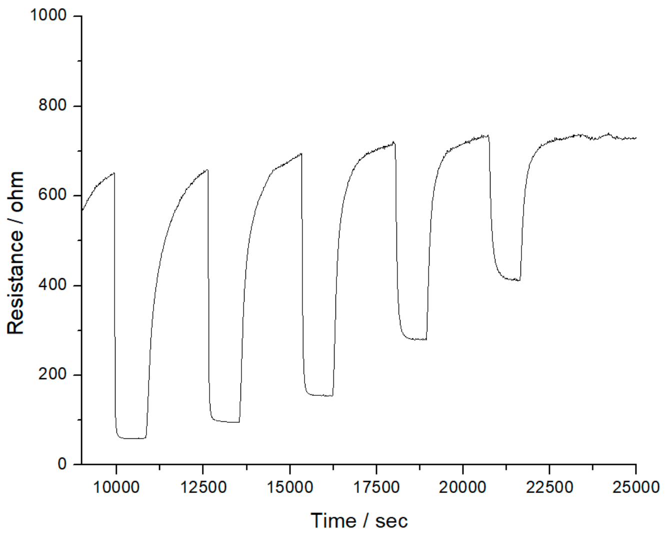

Figure S7. Dynamic response curves of Au-In_2O_3 cores-shell NPs subjected to Carbon monoxide (CO) gas at 375 °C toward 100-1 ppm gas.

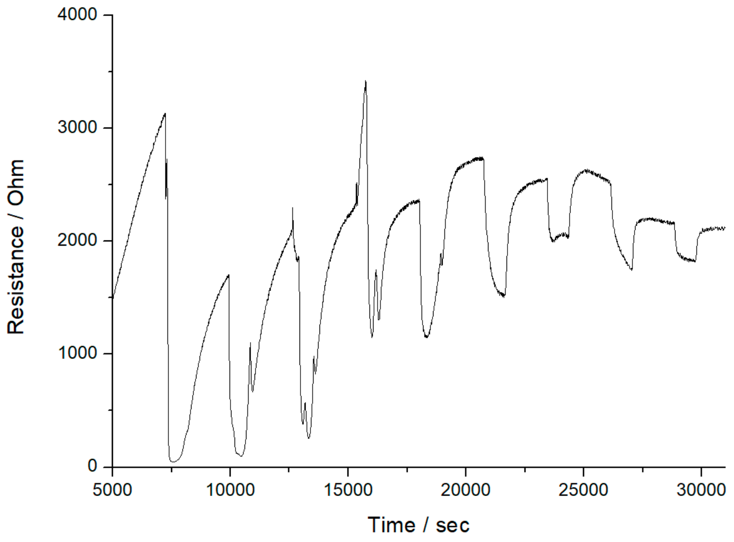

Figure S8. Dynamic response curves of Au-In_2O_3 cores-shell NPs subjected to NO_2 gas at 375 °C toward 100-1 ppm gas.

References

01. X. Li, X. Zhou, H. Guo, C. Wang, J. Y. Liu, P. Sun, F. Liu and G. Lu, ACS Appl. Mater. Interfaces, 2014, 6, 18661–18667.
02. Y.-S. Kim, P. Rai and Y.-T. Yu, Sens. Actuators, B, 2013, 186, 633–639.
03. T. T. D. Nguyen, D. V. Dao, D. –S. Kim, H. –J. Lee, S. –Y. Oh, I. –H. Lee, Y. –T. Yu, Effect of core and surface area toward hydrogen gas sensing performance using Pd@ZnO core-shell nanoparticles, J. Colloid Interface Sci. 587 (2021) 252–259.

04. H. –J. Lee, D. V. Dao and Y. –T. Yu, Superfast and efficient hydrogen gas sensor using PdAu alloy@ZnO core–shell nanoparticles, J. Mater. Chem. A, 8 (2020) 12968–12974.

05. Y. Xu, T. Ma, L. Zheng, L. Sun, X. Liu, Y. Zhao, J. Zhang, Rational design of Au/Co3O4-functionalized W18O49 hollow heterostructures with high sensitivity and ultralow limit for trimethylamine detection. Sens. Actuators B, 284 (2019) 202–212.

06. X.J. Liu, X.L. Sun, X.P. Duan, C. Zhang, K.R. Zhao and X.J. Xu, Core-shell Ag@In_2O_3 hollow hetero-nanostructures for selective ethanol detection in air Sens. Actuators B Chem., 305 (2020) 127450. S.M. Majhi, G.K. Naik, H.J. Lee, H.G. Song, C.R. Lee, I.H. Lee, Y.T. Yu

07. S.M. Majhi, G.K. Naik, H.J. Lee, H.G. Song, C.R. Lee, I.H. Lee and Y.T. Yu, Au@NiO core-shell nanoparticles as a p-type gas sensor: novel synthesis, characterization, and their gas sensing properties with sensing mechanism, Sens. Actuators B, 268 (2018) 223-231.

08. H.Y. Lee, J.H. Bang, S.M. Majhi, A. Mirzaei, K.Y. Shin, D.J. Yu, W. Oum, S. Kang, M.L. Lee, S.S. Kim and H.W. Kim, Conductometric ppb-level acetone gas sensor based on one-pot synthesized Au@Co_3O_4 core-shell nanoparticles, Sens. Actuators B, 359 (2022) 131550.

09. C.H. Wu, Z. Zhu, H.M. Chang, Z.X. Jiang, C.Y. Hsieh and R.J. Wu, Pt@NiO core-shell nanostructure for a hydrogen gas sensor, J. Alloy. Compd. 814 (2020) 151815.

10. Y.Y. Tian, L.L. Du, X.X. Xing, C. Wang, D.L. Feng, Z.X. Li and D.C. Yang, Surface-broken palladium and palladium oxide core/shell nanowires for stable hydrogen sensing at room temperature, Sens. Actuators B, 375 (2023) 132914.

11. S.K. Zhao, Y.B. Shen, P.F. Zhou, X.X. Zhong, C. Han, Q. Zhao and D.Z. Wei, Design of Au@WO_3 core-shell structured nanospheres for ppb-level NO_2 sensing, Sens. Actuators B. 282 (2019) 917-926.
